# Thinking Outside the Box: Developing Dynamic Data Visualizations for Psychology with Shiny

**DOI:** 10.3389/fpsyg.2015.01782

**Published:** 2015-12-01

**Authors:** David A. Ellis, Hannah L. Merdian

**Affiliations:** ^1^Department of Psychology, Lancaster UniversityLancaster, UK; ^2^School of Psychology, University of LincolnLincoln, UK

**Keywords:** visualization, knowledge-exchange, research methods, statistics, *R*, Shiny

## Abstract

The study of human perception has helped psychologists effectively communicate data rich stories by converting numbers into graphical illustrations and data visualization remains a powerful means for psychology to discover, understand, and present results to others. However, despite an exponential rise in computing power, the World Wide Web, and ever more complex data sets, psychologists often limit themselves to static visualizations. While these are often adequate, their application across professional psychology remains limited. This is surprising as it is now possible to build dynamic representations based around simple or complex psychological data sets. Previously, knowledge of HTML, CSS, or Java was essential, but here we develop several interactive visualizations using a simple web application framework that runs under the *R* statistical platform: *Shiny*. *Shiny* can help researchers quickly produce interactive data visualizations that will supplement and support current and future publications. This has clear benefits for researchers, the wider academic community, students, practitioners, and interested members of the public.

## Introduction

Psychological data analysis continues to develop with a recent shift in focus from significance testing to the exploration of effect sizes and confidence intervals (Schmidt, 1996; Sainani, 2009). At the same time, psychology and related fields have made meaningful contributions when it comes to developing innovative methods for visualizing and interpreting findings (for a brief history see Friendly, 2008). Historically, the focus has often been to maximize the expressive power of figures, both with regards to conveying the content and structure of the data as well as informing the analysis process (Campitelli and Macbeth, 2014; Marmolejo-Ramos, 2014). This has included a number of computational developments, such as the expansion of boxplots to include information about both distribution and density of the data (Marmolejo-Ramos and Matsunaga, 2009; Marmolejo-Ramos and Tian, 2010) or explorations of different data visualizations for particularly skewed data sets (Ospina et al., 2014).

**Figure 1:**
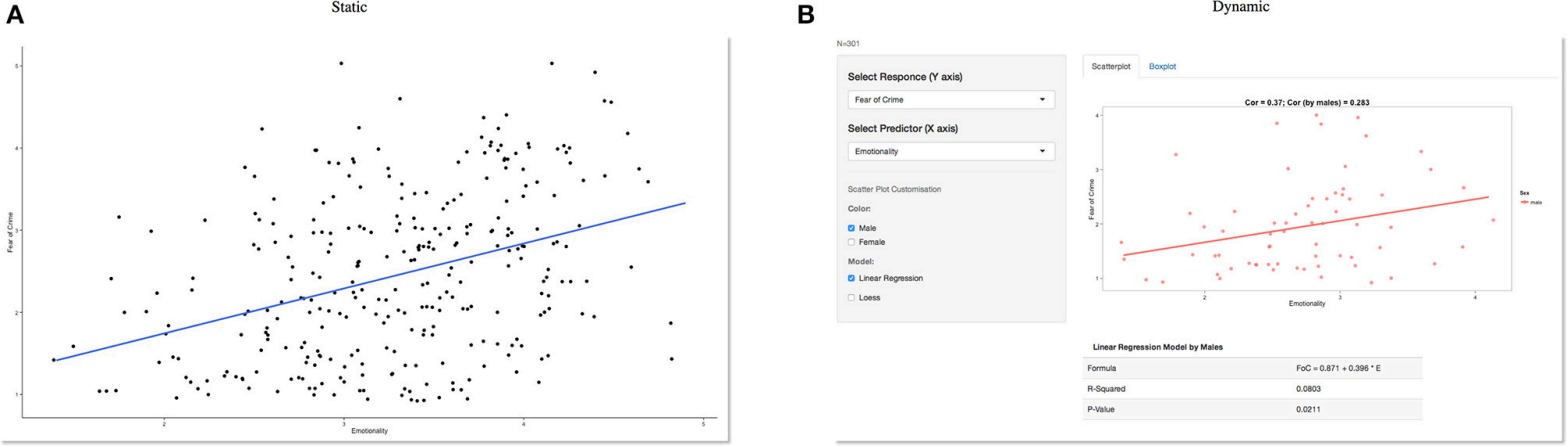
**Static vs. dynamic data visualization**. A static graph showing a positive relationship between fear and emotionality **(A)** can quickly be turned into a dynamic visualization **(B)** which in this example allows a website visitor to select a sub-group (male participants) of interest. Other variables are also available from the drop-down menus on the left and the included statistical analysis updates automatically based on user selections. However, this relies on the data being available to both a user interface and server to process these requests. Previously this was only possible by developing interactive web applications using a combination of HTML, CSS, or Java. However, this is no longer a limiting factor. For those who have a basic knowledge of *R*, the move from static to dynamic reporting is relatively straightforward.

However, while static graphical illustrations remain perfectly adequate in many instances, these have become problematic as we move toward larger and more complex data sets that evolve over time (Heer and Kandel, 2012). In a critical review concerning the use of data visualizations in scientific papers, Weissgerber et al. (2015) identified a number of limitations and misrepresentations linked to the current practice of using static figures when presenting continuous data from small sample sizes. Static data visualizations are also limited in the quantity and type of information that can be presented, which is typically directed toward the analysis conducted. These visualizations in isolation often raise additional questions about the data itself or suggest an alternative analysis. Dynamic representations on the other hand can provide an almost limitless supply of additional information; at a basic level, for example, this would enable a regression model to be re-calculated in real-time for male and female participants separately (Figure 1).

Complex applications can also provide online portals for interactive data augmentation and collaboration (Tsuji et al., 2014). However, such transformations rely on the data being available to both a user interface and server to process these requests. Previously this was only possible by developing interactive web applications using a combination of HTML, CSS, or Java, but this is no longer a limiting factor. For those who have a basic knowledge of *R*, the move from static to dynamic reporting is relatively straightforward (e.g., Xie, 2013).

Dynamic data visualization is likely to have clear advantages when teaching statistical concepts to undergraduate students; for example, Newman and Scholl (2012) pointed toward issues in students' interpretation of bar graphs (a static representation), with Moreau (2015) stating that visual and dynamic data representations may be more appropriate when teaching complex statistical concepts. For example, learning across multiple visual representations has been shown to improve students' understanding (Bodemer et al., 2004). It may also motivate students who were previously of the opinion that becoming statistically literate involves understanding numbers in isolation (Papastergiou, 2009).

Going further, dynamic data visualization can also fulfill the particular research needs of practitioners in the applied sciences including clinical and forensic psychology. One of the core competencies of professional psychologists in practice is to develop an understanding and application of scientific knowledge in evidence-based practice. These competencies should remain closely aligned to the development of methodological skills when evaluating research (e.g., American Psychological Association, 2011; British Psychological Society, 2014). Training is guided by the Scientist-Practitioner Model, postulating that effective psychological services are underpinned by research that is informed by questions arising from clinical practice (Jones and Mehr, 2007). However, there is no professional consensus in terms of the exact nature of the relationship between psychological science and professional practice (Peterson, 2000; Gelso, 2006). In their review of current issues regarding the future development of forensic psychology, Otto and Heilbrun (2002) emphasized practicing forensic psychology in line with the “relevant empirical data” (p. 16) but failed to systematically incorporate the scientific method as a development target for forensic psychologists. Gelso (2006) considers that a low level of research engagement by clinical doctorate graduates (e.g., Barlow, 1981; Peterson et al., 1982; Shinn, 1987) is due to neglect of the research training within the academic environment for professional psychologists, and to a lack of specific research skills required within their professions. Even for those undertaking pure research degrees, Aiken et al. (2008) identified significant gaps in the knowledge of doctoral students with major misunderstandings evident in statistics, measurement, and methodology training, specifically with regards to non-laboratory research, advanced research methods, and innovative methodology and research design. These training gaps constitute a particular disadvantage for clinical and forensic research productivity, where research is often based on single-case studies (e.g., ABA-designs in clinical practice) or small sample sizes (e.g., specific offender or clinical subtypes). Frequently, a large number of variables for each data point are available for a small number of cases that will often not fulfill the assumptions required for traditional linear tests (e.g., in offender profiling; Canter and Heritage, 1990s). Finally, with the introduction of mobile technology, applied field-research has the capacity to produce very large data sets through the use of mobile applications (e.g., in identifying friendship networks; Eagle et al., 2009; or displaying individual gait patterns; Teknomo and Estuar, 2014). However, both very small and very large data sets provide a challenge for standard linear representations and testing (Rothman, 1990), which we argue can in-part be compensated for with the use of dynamic data visualizations. This would also allow non-experts to repeat (complex) analyses in their own time, after the researcher has provided a summary (Valero-Mora and Ledesma, 2014).

At present, several barriers remain when integrating these methods with psychological research and practice. First, developing suitable applications that can process, analyze and visualize psychological data requires a significant allocation of resources. Second, the lack of concrete examples that directly relate to psychological data mean that current applications are often overlooked. In this tutorial paper, we aim to address both aspects by introducing *Shiny* (http://shiny.rstudio.com/), a data-sharing and visualization platform with low threshold requirements for most psychologists. We then provide several examples centered on a real-life forensic research dataset, which aimed to develop a predictive model for crime-related fear.

## Introducing shiny

*Shiny* allows for the rapid development of visualizations and statistical applications that can quickly be deployed online. By providing a web application framework for *R* (http://www.r-project.org), this platform allows researchers, practitioners and members of the public to interact with data in real-time and generate custom tables and graphs as required^1^.

Shiny applications have two components: a user-interface definition and a server script. These cleverly combine any additional data, scripts, or other resources required to support the application; data can either be uploaded to or retrieved from an online repository. The remainder of this paper will create and develop an interactive visualization using an example data set concerning factors that predict an individual's crime-related fear.

Developing any Shiny app or dynamic data visualization can be split into four steps:

- Data preparation
- Creating static content to guide development
- Development and testing
- Deploying an application online

### Data preparation

We recently collected data from around 300 participants which included a variety of variables that might predict an individual's fear of crime (see data.csv in Supplementary Material). While we were particularly interested in personality factors that predict fear, we also collected anxiety and well-being scores along with every participant's age and gender (see Table 1 for a list of included variables). We felt that that these findings may be of interest to members of the public and other interested parties (e.g., law enforcement agencies), and wanted to report the results in a dynamic fashion that allow external parties access the data and subsequent results.

**Table 1:** **Information about the included dataset—data.csv (Supplementary Material)**.

**Variable**	**Name in dataset**
Participant ID	Participant
Gender^*^	sex
Age	age
Victim of crime^*^	victim_crime
Honesty-humility	H
Emotionality	E
Extraversion	X
Agreeableness	A
Conscientiousness	C
Openness to experience	O
State anxiety	SA
Trait anxiety	TA
Happiness	OHQ
Fear of crime	FoC
Fear of crime (2 item version)	Foc2

Copies of this data set can be found in all included code folders (Supplementary Material).

* Categorical variable. Remaining variables are all numeric with higher scores indicating increased levels of each trait.

The included data set can be loaded into *R* using the read.csv command:

data <- read.csv("data.csv", header = T, sep = ",")

An identical dataset crime.csv is included with all example code folders.

Care should be taken by the data provider to only include variables that will be used as part of the final online application; for example, while almost all of our example variables were calculated from an extensive set of standardized measures, including the HEXACO-PI-R measure of personality (Ashton and Lee, 2009), we have not included the raw data for each measure to ensure that the final application will load and update quickly once online.

### Creating static content to guide development

Before creating any *Shiny* application, it is useful to experiment with some simple statistical analysis and static visualization in order to get a feeling for how the data can best be represented within an application. One may conclude that a static visualization (e.g., a single table or series of bar-graphs) is perfectly adequate without any additional development.

Code to install all relevant packages and generate static visualizations in *R* can be found in the static_graphics folder. From these examples, we concluded that for our data on crime-related fear, box and scatter plots were ideal when it came to exploring relationships between our variables of interest. Based on our original predictions, it became evident that specific aspects of personality, such as Emotionality, were likely to be the best predictors of crime-related fear. We also observed that there were a large number of variables and relationships we would like to explore and share with others; however, multiple scatter plots and regression lines would quickly become overwhelming, leading us to develop an application to share our results and data with others.

### Development and testing

We developed a series of examples that progress in complexity. Example 1 makes the simple transition from static to dynamic visualization using a Shiny function. Examples 2 and 3 add advanced customization features using additional graphical and statistical functions.

#### Example 1

To run the first example, load the *Shiny* library and set your working directory to the folder containing example1. This folder includes the data set and two scripts, ui.R and server.R (see below): library(“shiny”).

The move from static to dynamic visualization only requires a few additional lines of code. The ui.R script loads and labels the variables from the dataset. Here, we aimed to demonstrate how different personality factors might predict an individual's fear of crime, so these are labeled as responses and predictors accordingly. The second part of this script creates a simple *Shiny* page; various placeholders allow users to interact with the data. Finally, a command to print graphical output is placed at the end of this loop.

Moving to the server.R script, variable names defined within ui.R are replicated here. These variable names act as a link between both scripts. An *IF* function provides additional user interaction by differentiating between participants' gender. For example, if male, female or both genders are selected, then the chart will color each data point accordingly. If no participant gender is selected, then a standard plot is created that includes data from both male and female participants.

To run this example, simply type: runApp('example1') into the console. A scatter plot should now appear in a new window with a variety of options on the left (“Select Response,” “Select Predictor”). By experimenting with different predictors, the scatter plot will update accordingly; this process will assist the development of future predictions regarding what individual differences are more predictive of crime-related fear than others.

#### Examples 2 and 3^2^

**Figure 2:**
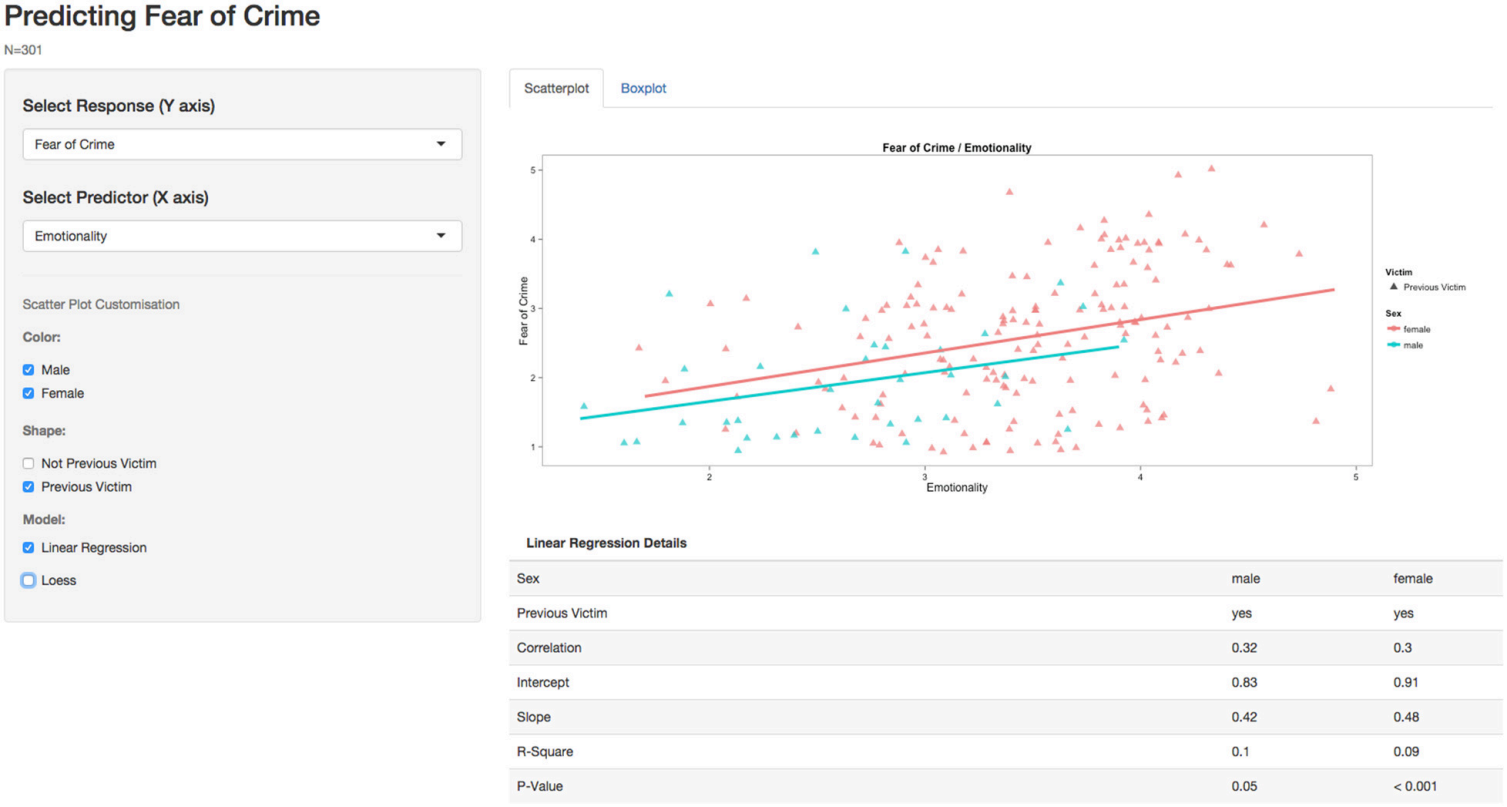
**Showing a variety of visualization options within Example 3**.

Examples 2 and 3 are developed directly from Example 1. Marked-up code is available in the Supplementary Material, example2 and example3. These can be run in an identical fashion to example1. Example 2 adds boxplots and statistical output, which again relies on standard graphical and mathematical functions in *R*. This version also allows the user to build linear regression models after choosing any predictor and response variable (e.g., the predictive value of Honest-Humility); statistical output is presented underneath the scatter plot, providing information relating to effect sizes and statistical significance. Box plots can be used to directly compare the distribution of scores on these variables, or to compare levels of crime-related fear between men and women directly. Example 3 (Figure 2) adds two additional functions, which handle a variety of potential visualization options. This provides separate regression outputs for male and female participants and/or those who have previously been a victim of crime.

### Deploying an application online^3^

There are several ways to deploy a *Shiny* application online; however, the fastest route is to create a Shiny account (http://www.shinyapps.io/) and install the *devtools* package by running the following code in your R console: install.packages('devtools').

Finally, the *rsconnect* package is also required and can be installed by running the following code in your R console: devtools::install_github('rstudio/rsconnect). Load this library: library(“rsconnect”). Once a shinyapps.io account has been created online and authorized, any of the included examples can quickly be deployed straight from the R console: deployApp(“example1”). However, it is also possible to host your own private *Shiny* server^4^.

Deployment of the application will allow anyone with an internet connection to engage with the data directly. However, the entire dataset could also be made available from the application itself with some additional development.

## Discussion

The last two decades have witnessed marked changes to the use and implementation of data visualizations. While research has often focused on the enhancement of existing static visualization tools, such as violin plots to express both density and distribution of data (Marmolejo-Ramos and Matsunaga, 2009), these remain limited due to their static nature. Specifically, static visualizations become exponentially more difficult to understand as the complexity of the content they aim to display increases (e.g., Teknomo and Estuar, 2014).

Such data-rich representations are likely to be helpful when teaching statistical concepts however, little research exists on its effectiveness within an educational context (Valero-Mora and Ledesma, 2014). While an expert user may believe they have created something practical and aesthetically pleasing, much of the literature surrounding human-computer interaction repeatedly demonstrates how a seemingly straightforward system that an expert considers “easy” to operate often poses significant challenges to new users (Norman, 2013). Future research is required in order to fully understand the effect interactive visualizations could have on a student's understanding of complex statistical concepts.

Dynamic visualizations remain a promising alternative to display and communicate complex data sets in an accessible manner for expert and non-expert audiences (Valero-Mora and Ledesma, 2014). The above worked examples demonstrate the straightforward and flexible nature of dynamic visualization tools such as *Shiny*, using a real-life example from forensic psychology. This move toward a more dynamic graphical endeavor speaks positively toward cumulative approaches to data aggregation (Braver et al., 2014), but it can also provide non-experts with access to simple and complex statistical analysis using a point-and-click interface. For example, through exploration of our fear of crime data set, it should quickly become apparent that while some aspects of personality do correlate with fear of crime, the results are not clear-cut when considering men and women in isolation and this may generate new hypotheses concerning gender differences and how a fear of crime is likely to be mediated by other variables.

While a basic knowledge of *R* is essential, dynamic visualizations can make a technically proficient user more productive, while also empowering students and practitioners with limited programming skills. For example, an additional *Shiny* application could automatically plot an individual's progress throughout a forensic or clinical intervention. Relationships between variables of improvement alongside pre and post scores across a several measures could also be displayed in real-time with results accessible to clinicians and clients. Dynamic data visualizations may therefore be the next step toward bridging the gap between scientists and practitioners.

The benefits to psychology are not simply limited to improved understanding and dissemination, but also feed into issues of replication. For example, the ability to compare multiple or pairs of replications side by side is now possible by providing suitable user interfaces. Tsuji et al. (2014), for example, have recently developed the concept of community-augmented meta-analysis (CAMA), which involves a combination of meta-analysis and an open repository (e.g., PsychFileDrawer.org; Spellman, 2012). These alone can improve research practices by ensuring that past research is integrated into current work. Using the intervention example from above, one can envision a further application that plots the progress of individual clients over several years, providing information on treatment change, outliers, and group trends over time.

In other areas of psychological research, much of this data already exists and the availability of data on open access repositories (e.g., such as *Dryad* or *Figshare*) makes data deposition in the first instance more straightforward. However, the advantages of open-access databases brings with it problems of navigation, organization and understanding. If these new developments are to reach their full potential and remain relevant to all psychologists, they still require a user-friendly interface that allows for rapid re-analysis and visualization. Of course, dynamic or interactive data visualizations are only going to become standard practice if psychologists use these methods on a regular basis. Researchers themselves will govern the speed of this development; journals may start to support this additional interactivity within publications. We hope that in addition to providing open access to data, psychologists will also popularize the shift toward dynamic visualizations in basic and applied research.

## Funding

A Research Investment Grant (RIF2014-31) from The University of Lincoln supported the preparation of this manuscript.

### Conflict of interest statement

The authors declare that the research was conducted in the absence of any commercial or financial relationships that could be construed as a potential conflict of interest.

## Footnotes

^1^An accompanying website is also available https://sites.google.com/site/psychvisualizations/

^2^Example 3 can be viewed online https://psychology.shinyapps.io/example3

^3^Additional instructions are available http://shiny.rstudio.com/articles/shinyapps.html

^4^http://www.rstudio.com/products/shiny/download-server/
